# Anesthetic technique and incidence of delirium after total knee or hip arthroplasty: a nationwide cohort study

**DOI:** 10.1186/s12871-024-02831-z

**Published:** 2024-11-27

**Authors:** Hey-ran Choi, Saeyeon Kim, In-Ae Song, Tak Kyu Oh

**Affiliations:** 1 Department of Anesthesiology and Pain Medicine, Inje University Sanggye Paik Hospital, Seoul, South Korea; 2 Department of Anesthesiology and Pain Medicine, Seoul National University Bundang Hospital, Gumi-ro 173 Beon-gil, Bundang-gu, Seongnam-si, 13620 South Korea; 3 Department of Anesthesiology and Pain Medicine, College of Medicine, Seoul National University, Seoul, South Korea

**Keywords:** Anesthesia, Arthroplasty, Cohort studies, Delirium

## Abstract

**Background:**

The optimal type of anesthesia for reducing postoperative delirium remains undetermined. This study aimed to assess the relationship between type of anesthesia and postoperative delirium.

**Methods:**

This retrospective national cohort study used data collected between 2016 and 2021 from the National Health Insurance Service of South Korea. Adult patients who underwent primary total hip or total knee arthroplasty under general or regional anesthesia were included. Patients with postoperative delirium were identified after arthroplasty according to the International Classification of Diseases 10th revision code for delirium (F05). The patients were divided into two groups: regional anesthesia (RA group) and general anesthesia (GA group). The primary endpoint was the incidence of postoperative delirium during hospitalization after total hip or knee arthroplasty.

**Results:**

Our study sample consisted of 664,598 patients: 474,932 in the RA group and 189,666 in the GA group. After propensity score (PS) matching, 276,582 patients (138,291 in each group) were included in the final analysis. In the PS-matched cohort, the incidence of delirium following total knee or total hip arthroplasty was 2.8% (3,842/138,291) in the GA group and 2.3% (3,147/138,291) in the RA group. In logistic regression, the RA group was associated with 18% (odds ratio: 0.82, 95% confidence interval: 0.78, 0.86; *P* < 0.001) lower postoperative incidence than the GA group.

**Conclusion:**

Compared to general anesthesia, regional anesthesia was associated with a decreased incidence of postoperative delirium in patients who underwent total hip or total knee arthroplasty. Our findings indicate that avoiding general anesthesia may prevent delirium after lower limb surgery.

**Supplementary Information:**

The online version contains supplementary material available at 10.1186/s12871-024-02831-z.

## Introduction

The incidence of osteoarthritis (OA) has been increasing with the continuous increase in global population age and obesity rates. Initially, patients benefited from traditional treatment approaches, such as pain medications, local injections, and physiotherapy. However, they eventually seek more effective treatments, such as total joint replacement surgery. OA affects the knees and hips, leading to a significant disease burden that often necessitates total joint replacement surgery [1].

A recent comprehensive study of 195 countries revealed that the estimated age-standardized prevalence rate of OA was 3754.2 individuals per 100,000 people. From 1990 to 2017, the global prevalence of hip and knee OA increased by 9.3% [2]. Despite the potential population decline due to lower fertility rates and reduced immigration, a continuous increase in both the incidence rate and total number of total knee and total hip arthroplasty (TKA and THA, respectively) procedures is predicted [3, 4]. Anesthesia plays a crucial role in joint arthroplasty in patients with OA experiencing aggravated functional impairments and pain. Depending on the patient’s health status, surgical location, and medical decisions made by the anesthesiologists and orthopedic surgeons, patients receive either general or regional anesthesia. As older adults constitute the majority of patients undergoing these surgeries, understanding the type of anesthesia that leads to fewer postoperative complications, especially delirium, is crucial. However, the optimal anesthetic method for reducing postoperative delirium remains unclear.

Previous studies exploring the relationship between anesthetic modalities and postoperative outcomes have yielded conflicting results. This study aimed to assess the incidence of delirium after primary TKA or THA in relation to the type of anesthesia, shedding light on this critical aspect of patient care.

## Methods

### Study design and ethical statement

We conducted a retrospective, nationwide cohort study using public data from the South Korean National Health Insurance Service (NHIS) database. The study protocol was approved by the Institutional Review Board (IRB) of Seoul National University Bundang Hospital (IRB) (IRB approval number: X-2303-819-902), and informed consent from the participants was waived by IRB of Seoul National University Bundang Hospital as we used anonymized data, retrospectively. The NHIS granted access to its public data and permitted the sharing of the following results (NHIS grant number: NHIS-2023-1-526). We strictly adhered to the Strengthening the Reporting of Observational Studies in Epidemiology guidelines [5].

### NHIS database

South Korean residents are registered under the NHIS, which is a public health insurance system. To receive compensation from the NHIS, physicians must submit accurate medical information related to patient treatment (including all procedures performed, drugs prescribed, and devices used) using the International Classification of Diseases 10th Revision (ICD-10) codes. Additionally, the NHIS contains demographic and socioeconomic data on residents, which are used to provide financial support through government subsidies.

### Study population

The research involved individuals aged 18 years and older who were hospitalized in South Korea and received TKA or THA from January 1, 2016, to December 31, 2021. In the analysis, only initial TKA or THA was considered for patients who underwent multiple TKA or THA procedures during the study period. Inclusion criteria were established to guarantee that the participants shared similar characteristics.

### Regional and general anesthesia

The patients were classified into two groups according to the type of anesthesia used for TKA or THA: one group received general anesthesia (GA group) and the other group received regional anesthesia (RA group). RA encompasses the use of spinal or epidural anesthesia or a combination of the two methods. Patients who developed RA and transitioned to GA during the course of treatment were categorized into the GA group. In addition, if local anesthesia or RA was administered in conjunction with GA, it was considered GA. When healthcare providers submit claims to the government for anesthesia services, they use distinct codes corresponding to each anesthetic technique. We collected information regarding the methods of anesthesia administered to the patients based on these billing codes. Even with the administration of a sedative during the procedure, the procedure was classified as regional anesthesia.

### Study endpoints

We investigated the incidence of postoperative delirium in patients hospitalized for TKA or THA between 2016 and 2021. ICD-10 code F05, which corresponds to the diagnosis of delirium, was used to accurately obtain the desired data from the NHIS database. Subsequently, we examined the relationship between anesthetic modality and the occurrence of postoperative delirium.

### Covariates

Data regarding patient demographics (age and sex) and socioeconomic status (employment status, household income level, and area of residence) at the time of hospital admission were obtained from the NHIS database. After excluding those enrolled in the Medical Aid Program, patients were divided based on quartile ratios. Residential areas were categorized as urban (Seoul or other metropolitan cities) or rural (all other areas). Additionally, we calculated the Charlson Comorbidity Index for each patient using the registered ICD-10 codes to evaluate comorbid disease status (Table S1). Preoperative psychiatric morbidity was considered a covariate because it is a known risk factor for delirium [6]. The psychiatric disorders included depression (F32, F33, and F34.1), schizophrenia (F20), suicide attempts or self-harm (X60–X84 and Y87.0), bipolar disorder (F31), anxiety disorder (F40 and F41), insomnia disorder (G47 and F51), and substance abuse (F10–F19). Based on the six grades of disability severity, patients were grouped into the severe disability group (patients with grades 1–3 disabilities) and the mild-to-moderate disability group (those with grades 4–6 disabilities). Because the government provides aid to patients with specific disability diagnoses and severity levels for their medical expenses and grants other social welfare benefits, both patients and physicians meticulously register related information in the NHIS database. Perioperative infusion of magnesium sulfate not only reduces the incidence of emergence agitation [7] but also provides pain relief, potentially reducing opioid requirements [8]. The duration of anesthesia for TKA and THA in hours was recorded and used as a covariate. Furthermore, we considered admission to the intensive care unit (ICU) and length of hospital stay. We also examined whether the patients received perioperative packed red blood cell transfusion, which is a known trigger of inflammation and a risk factor for postoperative delirium [9]. Hospitals were classified into four levels based on capacity, considering factors such as hospital type (tertiary general hospital, general hospital, or other) and the number of working physicians, specialist physicians, nurses, pharmacists, hospital beds, operating rooms, adult ICU beds, and emergency room beds.

### Statistical analysis

Continuous variables are described as means with SDs, and categorical variables are presented as numbers and percentages. To manage the factors linked to the hospital level, we used an agglomerative clustering approach to perform a hierarchical cluster analysis. Based on the findings of hierarchical clustering analysis, hospitals were categorized into four groups (Table S2).

Propensity score (PS) matching was used to mitigate the heterogeneity of each covariate between the RA and GA groups. This technique is recognized for its ability to reduce bias in observational studies [10]. In particular, PS matching was carried out without replacement, at a 1:1 ratio, and with a caliper width of 0.25 using the nearest-neighbor approach. After PS matching, the balance between the two groups was assessed using the absolute value of the standardized mean difference (ASD). If the ASD was less than 0.2, PS matching was considered appropriate.

In the PS-matched cohort, the association between the type of anesthesia and postoperative delirium was examined using logistic regression analyses, and the outcomes were presented as odds ratios (ORs) with 95% CIs. Multivariable logistic regression analyses were performed for sensitivity analysis to evaluate the generalizability of postoperative delirium findings from the PS-matched sample to the overall cohort. All factors were incorporated into the model for multivariable adjustment of the entire cohort. Additional sensitivity analysis using multivariable logistic regression was performed after excluding patients who died during hospitalization after TKA or THA because some patients could not be diagnosed with delirium due to death during hospitalization after TKA or THA. Furthermore, subgroup analyses were performed by grouping the patients according to the type of surgery (TKA or THA). R software (version 4.0.3; R Foundation for Statistical Computing, Vienna, Austria) was used for all statistical analyses, and *P* < 0.05 was considered statistically significant.

## Results

### Study population

**Fig. 1:**
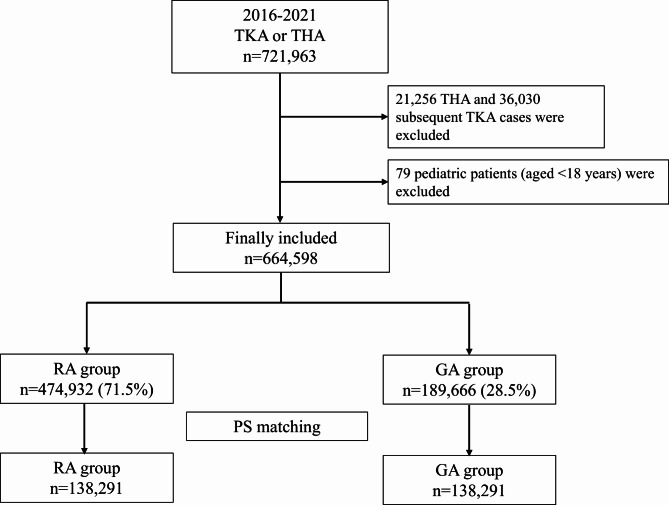
Flow chart depicting the patient selection process TKA, total knee arthroplasty; THA, total hip arthroplasty; RA, regional anesthesia; GA, general anesthesia; PS, propensity score

Between 2016 and 2021, 721,963 patients in South Korea will undergo TKA or THA. Of these, 664,598 adult patients were included in our analysis, after excluding those who underwent revision surgery and those younger than 18 years. Of the included patients, 474,932 (71.5%) and 189,666 (28.5%) belonged to the RA and GA groups, respectively (Fig. 1). After PS matching, 276,582 patients (138,291 in each group) were included in the final analysis. Table 1 shows the clinicopathological features of each group before and after PS matching. We checked for reduced heterogeneity of covariates between the two groups through PS matching (all ASDs were < 0.2 in the PS-matched cohort).

**Table 1:** Clinicopathological features of each group before and after PS matching

Variable	Before PS matching (*n* = 664,598)		ASD	After PS matching (*n* = 276,582)		ASD
	RA group *n* = 474,932	GA group *n* = 189,666		RA group *n* = 138,291	GA group *n* = 138,291	
Age, year	72.0 (8.7)	72.0 (10.4)	0.009	71.8 (10.1)	71.7 (10.3)	0.006
Male sex	97,484 (20.5)	43,879 (23.1)	0.052	31,906 (23.1)	31,406 (22.7)	0.032
Having a job	279,226 (58.8)	62,190 (32.8)	0.335	66,919 (48.4)	59,035 (42.7)	0.124
Household income level						
Q1 (lowest)	71,458 (15.0)	15,986 (8.4)		16,408 (11.9)	15,012 (10.9)	
Q2	65,737 (13.8)	14,725 (7.8)	0.197	15,053 (10.9)	13,808 (10.0)	0.037
Q3	96,538 (20.3)	21,123 (11.1)	0.235	21,801 (15.8)	19,900 (14.4)	0.045
Q4 (highest)	167,366 (35.2)	39,297 (20.7)	0.223	38,366 (27.7)	37,049 (26.8)	0.004
Medical aid program	31,265 (6.6)	8,204 (4.3)	0.144	7,351 (5.3)	7,401 (5.4)	< 0.001
Unknown	42,568 (9.0)	90,331 (47.6)	1.412	39,312 (28.4)	45,121 (32.6)	0.163
Residence						
Urban area	169,944 (35.8)	44,678 (23.6)		40,445 (29.2)	41,127 (29.7)	
Rural area	304,988 (64.2)	144,988 (76.4)	0.295	97,846 (70.8)	97,164 (70.3)	0.006
Underlying disability						
Mild to moderate	56,924 (12.0)	17,529 (9.2)	0.091	15,287 (11.1)	15,727 (11.4)	0.014
Severe	12,343 (2.6)	5,941 (3.1)	0.034	4,760 (3.4)	4,676 (3.4)	0.015
Preoperative psychiatric morbidity	64,452 (34.0)	188,179 (39.6)	0.112	50,466 (36.5)	49,384 (35.7)	0.006
CCI, point	0.9 (1.1)	0.8 (1.3)	0.111	0.9 (1.2)	0.9 (1.3)	0.048
Perioperative MgSO_4_ iv	2,926 (0.6)	786 (0.4)	0.021	1,358 (1.0)	723 (0.5)	0.078
Postoperative ICU admission	2,430 (0.5)	4,294 (2.3)	0.256	2,076 (1.5)	2,256 (1.6)	0.017
Duration of anesthesia in hour	2.4 (0.9)	2.0 (1.1)	0.443	2.4 (0.9)	2.2 (1.2)	0.165
Perioperative pRBC transfusion	297,357 (62.6)	98,199 (51.8)	0.228	79,100 (57.2)	74,201 (53.7)	0.077
Hospital level						
Level A	15,460 (3.3)	10,988 (5.8)		11,502 (8.3)	9,816 (7.1)	
Level B	190,086 (40.0)	23,277 (12.3)	0.561	34,674 (25.1)	22,978 (16.6)	0.173
Level C	55,539 (11.7)	72,712 (38.3)	0.858	44,016 (31.8)	47,864 (34.6)	0.070
Level D	213,847 (45.0)	82,689 (43.6)	0.036	48,099 (34.8)	57,633 (41.7)	0.154
Type of arthroplasty						
TKA	410,094 (86.3)	131,520 (69.3)		101,214 (73.2)	99,455 (71.9)	
THA	64,838 (13.7)	58,146 (30.7)	0.488	37,077 (26.8)	38,836 (28.1)	0.054
Year of surgery						
2016	74,424 (15.7)	24,176 (12.7)	0.087	22,131 (16.0)	19,785 (14.3)	0.073
2017	76,087 (16.0)	24,435 (12.9)	0.079	23,283 (16.8)	20,297 (14.7)	0.051
2018	78,014 (16.4)	27,397 (14.4)	0.063	21,800 (15.8)	21,669 (15.7)	0.011
2019	85,552 (18.0)	34,858 (18.4)	0.007	23,535 (17.0)	24,976 (18.1)	0.033
2020	78,555 (16.5)	37,761 (19.9)	0.089	23,015 (16.6)	25,001 (18.1)	0.049
2021	82,300 (17.3)	41,039 (21.6)	0.125	24,527 (17.7)	26,563 (19.2)	0.046

PS, propensity score; RA, regional anesthesia; GA, general anesthesia; ASD, absolute value of the standardized mean difference; CCI, Charlson comorbidity index; MgSO_4_, magnesium sulfate; ICU, intensive care unit; pRBC, packed red blood cell

### Analyses in PS-matched cohort

Table 2 presents the results of the PS-matched cohort analyses. After PS matching, the incidence of delirium after TKA and THA was 2.8% (3,842/138,291) in the GA group and 2.3% (3,147/138,291) in the RA group. In logistic regression, the RA group was associated with 18% (OR: 0.82, 95% CI: 0.78, 0.86; *P* < 0.001) lower postoperative incidence than the GA group.

**Table 2:** Analyses before and after PS matching

Variable	Event (*n*, %)	OR (95% CI)	*P*-value
Before PS matching			
GA group	6,592/189,666 (3.5)	1	
RA group	7,516/474,932 (1.6)	0.45 (0.43, 0.46)	< 0.001
After PS matching			
GA group	3,842/138,291 (2.8)	1	
RA group	3,147/138,291 (2.3)	0.82 (0.78, 0.86)	< 0.001

OR, odds ratio; CI, confidence interval; PS, propensity score; GA, general anesthesia; RA, regional anesthesia

### Analyses in the entire cohort

Table 3 presents the results of the multivariable logistic regression model for TKA or THA in the entire cohort as a sensitivity analysis. In multivariable model 1, the RA group had a 14% (OR: 0.86, 95% CI: 0.82, 0.89; *P* < 0.001) lower postoperative incidence than the GA group. After excluding 846 patients who died during hospitalization after TKA or THA, in multivariable model 2, the RA group was associated with 13% (OR: 0.87, 95% CI: 0.83, 0.90; *P* < 0.001) lower postoperative incidence than the GA group. All ORs with 95% CIs of the covariates in multivariable model 1 are presented in Table S3.

**Table 3:** Multivariable logistic regression for delirium after TKA or THA

Variable	OR (95% CI)	*P*-value
Covariate-adjusted model 1		
GA group	1	
RA group	0.86 (0.82, 0.89)	< 0.001
Covariate-adjusted model 2 after excluding patients who died during hospitalization after TKA or THA (*n* = 663,752)		
GA group	1	
RA group	0.87 (0.83, 0.90)	< 0.001

TKA, total knee arthroplasty; THA, total hip arthroplasty; OR, odds ratio; CI, confidence interval; GA, general anesthesia; RA, regional anesthesia

### Subgroup analyses

Table 4 summarizes the findings of subgroup analyses for each type of joint arthroplasty. In the TKA group, the RA group was associated with 17% (OR: 0.83, 95% CI: 0.78, 0.89; *P* < 0.001) lower postoperative incidence than the GA group. In the THA group, the RA group was associated with 14% (OR: 0.86, 95% CI: 0.81, 0.91; *P* < 0.001) lower postoperative incidence than the GA group.

**Table 4:** Subgroup analyses according to type of surgery

Variable	OR (95% CI)	*P*-value
**TKA group**		
GA group	1	
RA group	0.83 (0.78, 0.89)	< 0.001
**THA group**		
GA group	1	
RA group	0.86 (0.81, 0.91)	< 0.001

TKA, total knee arthroplasty; THA, total hip arthroplasty

## Discussion

In this nationwide retrospective study of adult patients undergoing primary TKA or THA, we discovered that patients who received regional anesthesia had a reduced risk of postoperative delirium compared with those who received general anesthesia. After excluding patients who died during hospitalization, the results were similar to those of the primary analysis. When we compared the patients based on the type of arthroplasty they underwent, the outcomes were comparable.

Previous studies reported varying outcomes regarding the association between anesthetic modalities and postoperative delirium following arthroplasty. Owing to differences in study designs and heterogeneity in anesthesia protocols among practitioners, the results remain inconclusive, although postoperative delirium may impair clinical outcomes in patients, particularly in vulnerable populations such as older adults. Although several meta-analyses have reported findings that differ from ours [11, 12], Memtsoudis et al. recently reported that neuraxial anesthesia is associated with a lower risk of postoperative delirium than general anesthesia [13].

The observed variations are likely attributable to the different designs employed in several studies. Scott et al. noted that the occurrence of delirium was marginally higher with the use of general anesthesia; however, this difference was not statistically significant (*P* = 0.651) [11]. The analysis employed data from 24 studies (a total of 2,895 patients) [11], which were significantly smaller than the sample size of our investigation. The use of a large amount of data, which enhanced the chances of statistical significance, could have led to this difference. Furthermore, the majority of previous studies were generic in nature [11, 12], looking for risk variables for postoperative delirium rather than using PS matching for a single anesthetic modality (RA vs. GA), as we did. As a result, caution must be exercised when interpreting the differences in outcomes.

Regional anesthesia is less likely to cause postoperative delirium than general anesthesia, which can be explained by two factors. First, regional anesthesia does not involve the loss of consciousness, whereas general anesthesia unintentionally causes oversedation because it requires amnesia, unconsciousness, and immobility. Postoperative delirium, a subgroup of delirium, is associated with reduced neuronal complexity, as indicated by a shift to lower-frequency activity on electroencephalographic (EEG) examinations [14]. Current research indicates a link between intraoperative burst suppression on EEG and postoperative delirium [15]. As fewer episodes of deep anesthesia (observed as burst suppression) are associated with reduced postoperative delirium [16], anesthesiologists are advised to monitor intraoperative EEG using processed EEG indices such as the bispectral index (BIS). However, despite the use of the BIS, oversedation under general anesthesia is unavoidable. BIS cannot accurately reflect EEG because it has a time delay and is affected by interference from electrical devices used in surgery, patient conditions (such as hypoglycemia and hypothermia), and the use of neuromuscular blockers [17].

In this context, a recent randomized clinical trial investigated whether EEG-guided general anesthesia to minimize EEG waveform suppression (excessive general anesthesia) reduced the incidence of delirium after cardiac surgery [18]. However, one study found that using EEG-guided anesthetic administration to reduce EEG suppression, in contrast to standard care, did not reduce the occurrence of postoperative delirium [18]. The type of surgery or sample size could potentially influence the outcomes, indicating a need for further investigation into this subject in the future.

Second, unlike regional anesthesia, general anesthesia causes neuroinflammation and disruption of the blood-brain barrier (BBB), resulting in a higher risk of postoperative delirium. Systemic anesthetic drugs required for general anesthesia activate the inflammatory cascade. The expression of pro-inflammatory cytokines and inflammatory mediators in the central nervous system (CNS) causes neuronal and synaptic dysfunction. Several studies have demonstrated a link between proinflammatory cytokines (C-reactive protein, interleukin-6, and tumor necrosis factor-alpha) and delirium. Neuroinflammatory alterations affect the BBB and alter neuronal excitability and synaptic transmission [19]. Several studies have found that anesthetics affect neuronal activity in a time- and dose-dependent manner, triggering neuroinflammation and disrupting CNS homeostasis. Propofol causes neurotoxicity and is associated with the destruction of BBB integrity owing to inflammation. In addition, endothelial cells exposed to propofol exhibit reduced resistance and greater permeability, indicating increased BBB permeability [20]. Furthermore, exposure to volatile inhalants such as sevoflurane and isoflurane has been linked to increased BBB permeability and neuroinflammation. Moreover, opioids alter the expression of tight junction proteins, which disrupts BBB integrity [21].

Our study had several limitations. First, we did not categorize the methods used to induce and maintain anesthesia. Anesthesiologists can select between total intravenous anesthesia and volatile inhalants for general anesthesia. Regional anesthesia techniques can be classified according to the specific types, locations, anesthetics, and adjuvants. Although we attempted to account for the use of perioperative magnesium sulfate infusion, a potent analgesic adjuvant, there are numerous alternative local and intravenous adjuvants. Second, data regarding postoperative analgesic use were not obtained. The use of patient-controlled analgesic devices or pain-relieving medications can also affect the incidence of postoperative delirium. Third, the NHIS database lacks sufficient information to collect data on certain critical characteristics, including alcohol use, smoking history, and body mass index. Fourth, various residual confounding factors may not have been considered, which may have affected our findings. Fifth, as our endpoint was delirium identified using an ICD-10 code during hospitalization, this study did not consider other lengths of hospital stay or the diagnostic systems implemented at each hospital. Finally, the potential impact of the sedatives used in the RA group, such as propofol, dexmedetomidine, and midazolam, was not considered, which could have affected the study outcomes.

## Conclusions

In conclusion, patients who underwent TKA or THA under regional anesthesia had a lower risk of postoperative delirium than those who underwent TKA or THA under general anesthesia. Our findings indicate that avoiding general anesthesia may prevent delirium after lower limb surgery.

## Electronic supplementary material

Below is the link to the electronic supplementary material.

Supplementary Material 1

Supplementary Material 2

Supplementary Material 3

Supplementary Material 4

## Data Availability

No datasets were generated or analysed during the current study.

## Abbreviations

CI: Confidence interval
GA: General anesthesia
ER: Emergency room
ICD-10: International Classification of Diseases, 10th Revision
ICU: Intensive care unit
IRB: Institutional Review Board
NHIS: National Health Insurance Service
OA: Osteoarthritis
OR: Odds ratio
RA: Regional anesthesia
SD: Standard deviation
TKA: Total knee arthroplasty
THA: Total hip arthroplasty
USD: United States dollar
