# Supplementary material for: Anesthetic technique and incidence of delirium after total knee or hip arthroplasty: a nationwide cohort study
Source: BMC Anesthesiol. 2024 Nov 27;24:433. doi: 10.1186/s12871-024-02831-z (PMC11600551; doi:10.1186/s12871-024-02831-z)
Supplement: Supplementary file 3 — Supplementary Material 3 [file 12871_2024_2831_MOESM3_ESM.docx]

Table S3. ORs with 95% CIs of all covariates in multivariable model 1

| Variable | | OR (95% CI) | *P*-value |
| --- | --- | --- | --- |
| Age, year | | 1.10 (1.10, 1.11) | <0.001 |
| Male sex | | 1.46 (1.40, 1.52) | <0.001 |
| Having a job | | 0.95 (0.91, 0.99) | 0.027 |
| Household income level | |  |  |
|  | Q1 (lowest) | 1 |  |
|  | Q2 | 1.10 (1.02, 1.19) | 0.019 |
|  | Q3 | 1.03 (0.96, 1.11) | 0.442 |
|  | Q4 (highest) | 1.04 (0.98, 1.11) | 0.205 |
|  | Medical aid program | 1.39 (1.28, 1.51) | <0.001 |
|  | Unknown | 1.51 (1.40, 1.62) | <0.001 |
| Residence | |  |  |
|  | Urban area | 1 |  |
|  | Rural area | 1.03 (0.99, 1.07) | 0.203 |
| Underlying disability | |  |  |
|  | Mild to moderate | 1.09 (1.03, 1.15) | 0.003 |
|  | Severe | 1.36 (1.25, 1.48) | <0.001 |
| CCI, point | | 1.11 (1.09, 1.12) | <0.001 |
| Postoperative ICU adission | | 1.19 (1.09, 1.29) | <0.001 |
| Perioperative MgSO4 infusion | | 1.08 (0.91, 1.29) | <0.386 |
| Perioperative transfusion | | 1.30 (1.25, 1.36) | <0.001 |
| Hospital level | |  |  |
|  | Level A | 1 |  |
|  | Level B | 2.85 (2.38, 3.43) | <0.001 |
|  | Level C | 5.34 (4.46, 6.38) | <0.001 |
|  | Level D | 4.46 (3.73, 5.33) | <0.001 |
| Type of arthroplasty | |  |  |
|  | TKA | 1 |  |
|  | THA | 2.84 (2.72, 2.96) | <0.001 |
| Year of surgery | |  |  |
|  | 2016 | 1 |  |
|  | 2017 | 1.03 (0.95, 1.11) | 0.510 |
|  | 2018 | 1.19 (1.10, 1.28) | <0.001 |
|  | 2019 | 1.28 (1.19, 1.38) | <0.001 |
|  | 2020 | 1.63 (1.52, 1.74) | <0.001 |
|  | 2021 | 1.96 (1.84, 2.10) | <0.001 |

OR, odds ratio; CI, confidence interval; CCI, Charlson comorbidity index; ICU, intensive care unit; TKA, total knee arthroplasty; THA, total hip arthroplasty
