# Supplementary material for: Anesthetic technique and incidence of delirium after total knee or hip arthroplasty: a nationwide cohort study
Source: BMC Anesthesiol. 2024 Nov 27;24:433. doi: 10.1186/s12871-024-02831-z (PMC11600551; doi:10.1186/s12871-024-02831-z)
Supplement: Supplementary file 4 — Supplementary Material 4 [file 12871_2024_2831_MOESM4_ESM.docx]

Table S3. All ORs with 95% CIs of the covariates in multivariable model 1

| Variable | | OR (95% CI) | *P*-value |
| --- | --- | --- | --- |
| Age, year | | 1.10 (1.10, 1.10) | <0.001 |
| Male sex | | 1.52 (1.46, 1.59) | <0.001 |
| Having a job | | 0.95 (0.91, 0.99) | 0.023 |
| Household income level | |  |  |
|  | Q1 (lowest) | 1 |  |
|  | Q2 | 1.11 (1.02, 1.20) | 0.011 |
|  | Q3 | 1.04 (0.96, 1.11) | 0.348 |
|  | Q4 (highest) | 1.05 (0.98, 1.12) | 0.160 |
|  | Medical aid program | 1.34 (1.23, 1.46) | <0.001 |
|  | Unknown | 1.51 (1.40, 1.63) | <0.001 |
| Residence | |  |  |
|  | Urban area | 1 |  |
|  | Rural area | 1.02 (0.97, 1.06) | 0.463 |
| Underlying disability | |  |  |
|  | Mild to moderate | 1.07 (1.01, 1.13) | 0.017 |
|  | Severe | 1.33 (1.23, 1.45) | <0.001 |
| CCI, point | | 1.08 (1.07, 1.09) | <0.001 |
| Preoperative psychiatric morbidity | | 1.89 (1.82, 1.96) | <0.001 |
| Postoperative ICU adission | | 1.17 (1.08, 1.27) | <0.001 |
| Perioperative MgSO4 infusion | | 1.10 (0.92, 1.32) | 0.285 |
| Perioperative transfusion | | 1.30 (1.25, 1.36) | <0.001 |
| Duration of anesthesia in hour | | 0.93 (0.91, 0.95) | <0.001 |
| Hospital level | |  |  |
|  | Level A | 1 |  |
|  | Level B | 2.43 (2.03, 2.93) | <0.001 |
|  | Level C | 5.04 (4.21, 6.03) | <0.001 |
|  | Level D | 3.81 (3.18, 4.56) | <0.001 |
| Type of arthroplasty | |  |  |
|  | TKA | 1 |  |
|  | THA | 2.61 (2.51, 2.72) | <0.001 |
| Year of surgery | |  |  |
|  | 2016 | 1 |  |
|  | 2017 | 1.07 (0.99, 1.16) | 0.090 |
|  | 2018 | 1.27 (1.18, 1.37) | <0.001 |
|  | 2019 | 1.39 (1.30, 1.50) | <0.001 |
|  | 2020 | 1.79 (1.67, 1.91) | <0.001 |
|  | 2021 | 2.13 (1.99, 2.28) | <0.001 |

OR, odds ratio; CI, confidence interval; CCI, Charlson comorbidity index; MgSO_4_, magnesium sulfate; ICU, intensive care unit; pRBC, packed red blood cell; TKA, total knee arthroplasty; THA, total hip arthroplasty
